# Nano- and Micro-Patterned S-, H-, and X-PDMS for Cell-Based Applications: Comparison of Wettability, Roughness, and Cell-Derived Parameters

**DOI:** 10.3389/fbioe.2018.00051

**Published:** 2018-05-01

**Authors:** Marina Scharin-Mehlmann, Aaron Häring, Mathias Rommel, Tobias Dirnecker, Oliver Friedrich, Lothar Frey, Daniel F. Gilbert

**Affiliations:** ^1^Chair of Electron Devices, Friedrich-Alexander-Universität Erlangen-Nürnberg, Erlangen, Germany; ^2^Institute of Medical Biotechnology, Friedrich-Alexander-Universität Erlangen-Nürnberg, Erlangen, Germany; ^3^Fraunhofer Institute for Integrated Systems and Device Technology (IISB), Erlangen, Germany; ^4^Erlangen Graduate School in Advanced Optical Technologies (SAOT), Erlangen, Germany

**Keywords:** nano- micrometer-patterned PDMS, wettability, AFM, water contact angle measurement, HFF-1 cells, cell viability, cell morphology

## Abstract

Polydimethylsiloxane (PDMS) is a promising biomaterial for generating artificial extracellular matrix (ECM) like patterned topographies, yet its hydrophobic nature limits its applicability to cell-based approaches. Although plasma treatment can enhance the wettability of PDMS, the surface is known to recover its hydrophobicity within a few hours after exposure to air. To investigate the capability of a novel PDMS-type (X-PDMS) for *in vitro* based assessment of physiological cell properties, we designed and fabricated plane as well as nano- and micrometer-scaled pillar-patterned growth substrates using the elastomer types S-, H- and X-PDMS, which were fabricated from commercially available components. Most importantly, we compared X-PDMS based growth substrates which have not yet been investigated in this context with H- as well as well-known S-PDMS based substrates. Due to its applicability to fabricating nanometer-sized topographic features with high accuracy and pattern fidelity, this material may be of high relevance for specific biomedical applications. To assess their applicability to cell-based approaches, we characterized the generated surfaces using water contact angle (WCA) measurement and atomic force microscopy (AFM) as indicators of wettability and roughness, respectively. We further assessed cell number, cell area and cellular elongation as indirect measures of cellular viability and adhesion by image cytometry and phenotypic profiling, respectively, using Calcein and Hoechst 33342 stained human foreskin fibroblasts as a model system. We show for the first time that different PDMS types are differently sensitive to plasma treatment. We further demonstrate that surface hydrophobicity changes along with changing height of the pillar-structures. Our data indicate that plane and structured X-PDMS shows cytocompatibility and adhesive properties comparable to the previously described elastomer types S- and H-PDMS. We conclude that nanometer-sized structuring of X-PDMS may serve as a powerful method for altering surface properties toward production of biomedical devices for cell-based applications.

## Introduction

Cellular physiology and viability as well as the cells' ability to migrate, to proliferate or to interact with an artificial growth surface *in vitro* or the extracellular matrix (ECM) *in vivo*, strongly depend on the properties of local structural and physical characteristics, including wettability, three-dimensional topography and stiffness (Discher et al., 2005; Yim et al., 2005; Bettinger et al., 2009; Martínez et al., 2009; Hwang et al., 2010; Ranella et al., 2010; Dowling et al., 2011; Rupp et al., 2014; Song and Ren, 2014; Keshavarz et al., 2016; Moyen et al., 2016). Artificial growth surfaces, commonly used for maintaining cells *in vitro*, are mostly two-dimensional substrates which are optimized with regard to high wettability, i.e., hydrophilicity that facilitates cellular adhesion and establishment of monolayer cultures (Cox et al., 2002; Yamamoto et al., 2014; Guan et al., 2015). However, cells in monolayer cultures lack the complex three-dimensional environment of the ECM in native tissues, including nano- to micrometer sized structures such as collagen fibrils, and may not recapitulate what is observed *in vivo* (Doyle et al., 2013; Wrzesinski and Fey, 2015; Gilmour et al., 2016; Sánchez-Romero et al., 2016). In fact, there is no doubt, that cells grown on artificial two-dimensional surfaces exhibit strongly altered physiological properties compared to the same cells maintained in native ECM (Cukierman et al., 2001; Ghosh and Ingber, 2007; Green and Yamada, 2007; Chambers et al., 2014; Sánchez-Romero et al., 2016; Young and Reed, 2016). Thus, for *in vitro*-based assessment of the physiological properties of cells *in vivo*, improved platforms for biomedical applications, such as lab-on-a-chip devices, providing an artificial nano- to micrometer-structured environment that mimics a patterned topography of the native ECM, are urgently required (Li and Kilian, 2015).

The silicone elastomer polydimethylsiloxane (PDMS) has been a long-serving material for manufacturing systems for biomedical applications as it is biocompatible, robust, cost-effective and simple to handle and is considered well-suited for the development of lab-on-a-chip devices (Peterson et al., 2005; Millet and Gillette, 2012; Zhou et al., 2012; Halldorsson et al., 2015). Depending on its Young's modulus or stiffness, PDMS may be assigned to one of the three types S-, H-, and X-PDMS (Verschuuren, 2010), which are differently amenable to generating nano- to micrometer-patterned matrices and thus, mimicking the three-dimensional topography of the native ECM. Soft or S-PDMS (e.g., Sylgard® 184, Dow Corning Corporation) is the most commonly used type and has proven useful for a variety of biomedical applications, including tissue engineering and microfluidics systems. However, its applicability for the production of structured, and in particular nanometer-scaled PDMS matrices, is limited due to its low stiffness and Young's modulus (2–3 MPa) (Verschuuren, 2010). H-PDMS is a more rigid PDMS type with a comparably higher Young's modulus (8–12 MPa). This material has proven to be suitable for generating sub-micrometer-patterned structures, however, it has also shown to be unsuitable for fabricating nanometer-sized structures with high aspect ratios (Verschuuren, 2010; Schmitt et al., 2012; Scharin et al., 2014). X-PDMS is the stiffest of the three PDMS types with a Young's modulus of 20–80 MPa (Verschuuren, 2010). X-PDMS has proven suitable for the production of structures in the nanometer range with high accuracy and reproducibility (Verschuuren, 2010).

Despite its general versatility and suitability for the generation of artificial nano- to micrometer-patterned ECM-like structures, PDMS is highly hydrophobic and thus, rather inappropriate for cell-based applications (Zhou et al., 2012; Zilio et al., 2014; Jellali et al., 2016). Due to the fact that cell adhesion is favored on hydrophilic substrates, which exhibit high wettability and small contact angles, typically in the range between 0 and 90°, PDMS requires chemical or physical treatment prior to application in cell-based approaches (Wei et al., 2007; Gittens et al., 2014; Rupp et al., 2014). Plasma treatment is suitable to enhance the wettability of PDMS growth surfaces. However, it has been reported that the positive effect is of short duration and that hydrophobicity recovers within a few hours of exposure to ambient air (room temperature and laboratory humidity conditions) (Owen and Smith, 1994; Fritz and Owen, 1995; Hillborg and Gedde, 1998; Bhushan et al., 2008; Bodas et al., 2008; Gomathi et al., 2008; Ambrosia et al., 2013; Scharin et al., 2014). Modification of the surfaces' roughness is another approach for altering the wettability of PDMS substrates (Owen and Smith, 1994; Hillborg and Gedde, 1998). The interplay between surface roughness or geometry and wettability can be described by two popular models: the Wenzel as well as the Cassie and Baxter model (Wenzel, 1936; Bhushan and Jung, 2006; Quéré, 2008). In the Wenzel model that implies a homogeneous surface, it is predicted that the initial state is amplified along with increasing roughness, i.e., hydrophilic and hydrophobic surfaces turn into even more hydrophilic and hydrophobic surfaces, respectively (Wenzel, 1936; Quéré, 2008). The Cassie and Baxter model assumes a heterogeneous surface with multiple interfaces, e.g., a droplet-air- or droplet-bulk material-interface. This model predicts that the wettability is determined by surface topography (roughness and surface pattern) regardless of the initial state of the surface (Bhushan and Jung, 2006; Bhushan et al., 2008; Quéré, 2008).

In the present study, we evaluated the characteristics and applicability of nano- and micrometer-patterned PDMS growth substrates for the development of e.g., lab-on-a-chip devices with improved ECM-like properties and compared them with results on plane PDMS growth substrates. To this end, we aimed to manufacture various growth substrates using the elastomer types S-, H-, and X-PDMS, and to alter their wettability (i) by plasma treatment and (ii) based on nano- and micrometer scaled three-dimensional surface structuring. Plasma treatment was used to investigate its influence on the wettability of all PDMS types with emphasis on comparing results for X-PDMS being studied for the first time in this context with results from H-PDMS and S-PDMS characterized before (Scharin et al., 2014). We further aimed to analyse the roughness of the manufactured PDMS growth substrates using atomic force microscopy (AFM) and to measure the contact angle of water on the fabricated surfaces as an indicator of wettability. To evaluate the different PDMS substrates with respect to their applicability to cell-based approaches, we intended to assess cell number, cell area and cellular elongation as measures of cellular viability and adhesion by image cytometry and phenotypic profiling, respectively, using Calcein and Hoechst 33342 stained HFF-1 cells (human foreskin fibroblasts) as a model system. We aimed to assess the cell number in our experiments because in cell-based high-content and high-throughput screening, using, e.g., large-scale genetic or chemical libraries, determination of the number of cells has been a long-serving, robust and straightforward indicator of cellular viability (Gilbert et al., 2011; Gilbert and Boutros, 2016; Schneidereit et al., 2016). We decided to use human foreskin fibroblasts as these cells are characterized by a strong, elongated morphotype, when maintained in monolayer cultures and also because fibroblasts have previously been applied for characterization of PDMS growth surfaces (van Kooten et al., 1998; Stanton et al., 2014). The cellular shape in *in vitro* cultures is an important estimator of cellular physiology and viability and can serve as an indicator of how strong a cell is attached to a growth substrate (Galluzzi et al., 2007; Barnhart et al., 2011; Dakhil et al., 2016; Kuenzel et al., 2016). For adherent cell lines, a round shape, unless during cell division, typically reflects altered cell fitness and/or adhesion to a growth substrate whereas an elongated morphology may indicate a healthy state and unaltered attachment to the culture surface (Stanton et al., 2014; Gilbert et al., 2016). The cell area has previously been associated with cellular adherence on PDMS growth surfaces and has thus also been assessed in our study (Barnhart et al., 2011; Wu et al., 2013; Stanton et al., 2014).

For phenotypic profiling as well as for assessment of the cell number as a measure of cell viability, we intended to label HFF-1 cells with the fluorescent indicators Calcein acetoxymethyl (AM) and Hoechst 33342. Both markers are being commonly applied indicators to assess cellular viability (Larsson and Nygren, 1989; Braut-Boucher et al., 1995; Gilbert et al., 2011; Menzner et al., 2015; Gilbert and Boutros, 2016). Upon permeation of the cell membrane, non-fluorescent Calcein-AM is hydrolyzed by non-specific intracellular esterases and the product Calcein, a hydrophilic, strongly fluorescent molecule remains inside the cell. Hoechst 33342 exhibits distinct fluorescence emission upon binding into the minor groove of DNA.

## Materials and methods

### Elastomer fabrication

The preparation and fabrication of elastomer substrates has been conducted in a clean environment, i.e., in the clean room. S-PDMS was prepared by mixing the two-components of Sylgard 184 (Dow Corning), silicone base and curing agent in 10:1 mass ratio as indicated in the manufacturer's instructions.

H-PDMS was prepared by mixing two components (A and B) in a 1:0.3253 mass ratio. Component A was prepared by mixing trimethylsiloxy terminated vinylmethylsiloxane-dimethylsiloxane (VDT-731; Gelest, Inc.), tetramethyl-tetramethyl-disiloxane (Fluka 87927; Sigma-Aldrich Co. LLC.), and platindivinyl-tetramethyldisiloxane (SIP 6831.1; Gelest, Inc.) in equal parts. Component B was prepared from methylhydrosiloxane-dimethylsiloxane (HMS-301; Gelest).

X-PDMS was prepared equivalently to H-PDMS (A and B mass ratio 1:0.31283) with the difference that component A was prepared by mixing VDT-731 (Gelest, Inc.), SIP 6831.1 (Gelest, Inc.), tetravinyl-tetramethyl-cyclotetrasiloxane (SIT-7900.0) and vinyl Q-siloxanes in xylene VQX-221 (Gelest, Inc.).

Plane as well as nano- and micrometer-scaled pillar-patterned S-, H-, and X-PDMS growth substrates were fabricated as described in our previous work (Scharin et al., 2014). The patterned growth substrates were prepared from Si masters with nominal hole diameter and pitch of 2 and 6 μm, respectively, and nominal hole depths between 130 and 1,800 nm. For plane PDMS samples, a non-structured plane Si wafer was used.

For S-PDMS samples the degassed S-PDMS was poured onto a 150 mm silicone wafer which was fixed in a commercially available replication tool (Süss). The replication tool consists of a vacuum holder for the silicone wafers, a moveable closure head, which has vacuum holders for a glass plate, and adjustable screws, in order to lower the closure head in a defined manner. The wafer and glass plate holder were heated to 50°C. S-PDMS was poured onto the wafer and the closure head was carefully lowered until the glass plate was in contact with the S-PDMS. After the initial contact of glass plate and S-PDMS the closure head was further lowered until the S-PDMS was completely spread on the wafer. Due to the adjustable screws the thickness of the S-PDMS can be controlled. After 24 h of curing at 50°C the silicone wafer with the S-PDMS was removed from the replication tool and the S-PDMS sample was peeled off from the wafer. The demolded S-PDMS exhibited a thickness of around 800 μm. The H- and X-PDMS samples were also produced in the replication tool. However, due to the brittleness of H- and X-PDMS, we produced two layer systems with a thick S-PDMS layer to ensure proper handling of PDMS samples. This was realized by first spin coating H- or X-PDMS onto the wafer, which was then placed into the replication tool and a layer of S-PDMS was poured on top of the H- or X-PDMS. These H- and X-PDMS samples were cured for 72 h at 50°C before being removed from the replication tool and peeled off the wafer. The thickness of the two layer systems amounted to approximately 850 μm, i.e., the thickness of the H- or X-PDMS layer was approximately 50 μm.

After fabrication of the wafer scale growth substrates circular PDMS substrates (14 mm diameter) were cut out for cell experiments. Always one substrate from the same growth substrate was used for physical analysis [i.e., AFM, water contact angle (WCA) measurements, and scanning electron microscopy in this order] and other substrates were used for cell experiments. Prior to cell-based experiments, samples were washed several times in ethanol and subsequently with water and culture medium. Prior to AFM, SEM, and WCA measurements, samples were cleaned by sonification and were dried using compressed air.

### Mechanical properties of the elastomers

For identically prepared test samples, Young's modulus was measured by pico-indentations using an AFM and a nano-indenter (Bruker ICON). Each sample was measured at least 6 times.

### Plasma treatment

The wettability of PDMS substrates was altered by plasma treatment using low frequency plasma (40 kHz) in a PlasmaPrep2 tool (GaLa Instrumente). Plane and pillar-patterned PDMS films were treated for 10 min with forming gas plasma (95% N_2_/5% H_2_, 400 cm^3^/min, 150 W plasma power), since PDMS surfaces treated by N_2_/H_2_ plasma have shown super-hydrophilic behavior in previous studies (Scharin et al., 2014). Immediately after plasma treatment samples were stored in distilled water to minimize hydrophobic recovery (Scharin et al., 2014).

### Quantification of water contact angle

The wettability, i.e., the hydrophilicity or hydrophobicity of the fabricated and differently treated PDMS surfaces was determined by quantification of the WCA (α) of a sessile drop of ultrapure water (MilliQ) using an OCA 30 device (Data Physics Instruments GmbH). For PDMS samples which were modified by N_2_/H_2_ plasma, all measurements were performed within 10 min after surface treatment. Ten measurements with distilled water drops were carried out on each PDMS sample.

### Scanning electron microscopy

The topography of manufactured PDMS substrates was qualitatively analyzed by scanning electron microscopy using a Helios Nanolab 600 Dual-Beam system (FEI) (working distance: 4.2 mm, acceleration voltage: 10 kV, magnification: 3,500x).

### Atomic force microscopy

The surface roughness of the samples was determined by AFM using a Dimension 5000 atomic force microscope (Bruker) in tapping mode. For plane and flat pillar patterned PDMS (130 and 190 nm nominal pillar height) surfaces, a standard tapping mode AFM probe (NCHR, Nanoworld) was used. For patterned surfaces with pillars of 1,800 nm height high-aspect-ratio AFM probes (AR5T-NCHR, Nanoworld) were used. The scanning area was 50 × 50 μm^2^, the scanning rate 0.5 Hz. In this scanning area each roughness value (root mean square roughness R_q_) was evaluated from five 10 × 10 μm^2^ areas.

### Reagents

All reagents were obtained from Sigma-Aldrich if not stated otherwise. Calcein-AM and Hoechst 33342 were prepared as 10 and 100 mM stocks, respectively, in dimethylsulphoxide (DMSO). All stocks were frozen at −20°C. From these stocks, solutions for experiments were prepared on the day of experiments.

### Cell line

HFF-1 cells (SCRC-1041™) were purchased from The American Type Culture Collection (ATCC).

### Cell culture

All experiments were performed with HFF-1 cells cultured in Dulbecco's modified Eagle's medium (DMEM, Invitrogen) supplemented with 10% fetal calf serum and penicillin (100 U/ml)/streptomycin (100 mg/ml) (Sigma-Aldrich). Cells were cultured at 37°C, 5% CO_2_ in a humidified incubator according to standard procedures and were passaged weekly.

### Preparation of PDMS substrates for experiments

**Figure 1:**
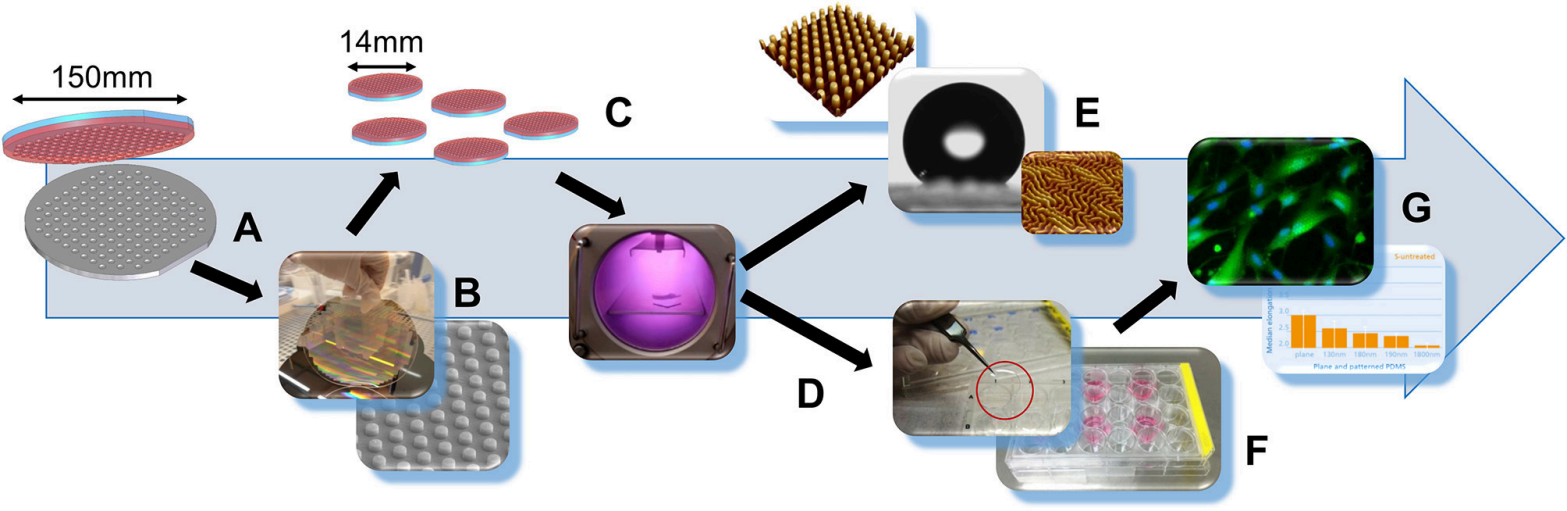
Workflow of substrate fabrication and experimentation. **(A)** S-, H-, and X-PDMS fabrication using plane or patterned silicone master wafer. **(B)** Separation of a 150 mm diameter PDMS thin film from master wafer with scanning electron microscopy image of a PDMS surface with pillars. **(C)** Punching-out of circular 14 mm PDMS films. **(D)** Plasma treatment of plane PDMS foils. **(E)** Surface characterization by water contact angle measurement and atomic force microscopy for evaluation of wettability and roughness. **(F)** Placing of circular PDMS substrates into cell culture plates. **(G)** Cell-based experimentation using human foreskin fibroblasts (HFF-1 cells); assessment of cell number, cell area and elongation factor as measures of viability and cellular adhesion for estimation of the applicability to cell-based approaches.

The circular PDMS substrates (14 mm diameter) were placed into 24-well plates (TPP, Switzerland) (see Figure 1F). To prevent upfloating in culture medium, PDMS growth substrates were fixed at the bottom using custom-designed and 3D printed acrylonitrile butadiene styrene (ABS) clamps (not shown). PDMS foils were placed in columns 1–5. As a preparatory step prior to culturing cells on growth substrates, the PDMS foils were sterilized in 70% ethanol and were washed twice in Dulbecco's modified Eagle's medium (DMEM, Invitrogen) supplemented with 10% fetal calf serum and penicillin (100 U/ml)/streptomycin (100 mg/ml).

### Preparation of cells for experiments

The day before imaging experiments, 5 × 10^4^ cells were seeded into each well of a 24-well plate containing sterilized and washed PDMS foils and were cultured at 37°C, 5% CO_2_ in a humidified incubator (for 24 h). The next day and approximately 1 h prior to commencement of experiments, the culture medium was removed and was replaced by standard imaging solution containing (in mM): NaCl 140, KCl 5, CaCl_2_ 2, MgCl_2_ 1, HEPES (4-(2-hydroxyethyl)-1-piperazineethanesulfonic acid) buffer solution 10, and glucose 10 (pH 7.4, adjusted with NaOH) and supplemented with 10 μM Calcein-AM and 10 μM Hoechst 33342. With this staining solution cells were incubated for 1 h at 37°C, 5% CO_2_. Upon fluorescence labeling the staining solution was entirely removed from the wells and was replaced by pure standard imaging solution. The standard imaging solution also referred to as extracellular solution, decreases background light as compared to culture medium and thus, allows an optimal signal-to-noise ratio for imaging cells using fluorescence microscopy. Immediately after solution exchange, cells were imaged using the high-content imaging system described below.

### Imaging experiments

The 24-well plate was placed onto the motorized stage of a high-end imaging system (Nikon Eclipse Ti, Nikon, Japan), and cells were imaged with a 10x objective (CFI Plan Fluor DL 10X Phase, N.A. 0.30, Nikon, Japan). Illumination from a Xenon lamp (Lambda LS, Sutter Instruments, USA), passing through a filter block (Calcein: EX 465-495, DM 505, BA 515-555; Hoechst 33342: EX 340-380, DM 400, BA 435-485, Olympus, Japan) was used to excite and detect Calcein and Hoechst 33342 fluorescence signals. Fluorescence was imaged by a sCMOS camera (NEO, Andor, Ireland) and digitized to disk onto a personal computer (Dell Precision T3500, Dell, USA). The primary resolution of the camera was 2,560 × 2,160 pixel, although images were binned (2 × 2), resulting in a resolution of 1,280 × 1,080 pixel.

### Image analysis

Images were quantitatively analyzed using a modified version of DetecTiff^©^ software (Gilbert et al., 2009). In brief, fluorescence images were segmented using an iterative size and intensity-based thresholding algorithm, and the number of cells (*Cells per image*), the cell area (*Area*, in pixels) as well as the elongation factor (*Elongation factor*) of each cell were calculated. The elongation factor is defined as

$$\begin{array}{l} Elongation\;factor=\frac{Max.\;intercept}{Mean\;perpendicular\;intercept} \end{array}$$

Where *Max. intercept* is the length of the longest segment and *Mean perpendicular intercept* is the perpendicular mean length of the chords in an object. The elongation factor of a perfectly circular object is 1, whereas an elongated and stretched-out object is represented by a value > 1 or >> 1.

### Data analysis

For the morphological descriptors *Area* and *Elongation factor*, single cell-derived data from a total of 8–12 images per experiment were pooled and the mean value and standard error of the mean SEM were calculated. The value *Cells per image* was averaged from a total of 8–12 images. Data were processed using MS Office 2013, SigmaPlot, Origin 7G, Labview 2013 and ImageJ. Statistical analysis was done based on two-way and three-way ANOVA tests followed by pairwise multiple comparison based on the Holm-Sidak method. Normality and homogeneity of the variances was tested based on the Kolmogorov-Smirnov or Shapiro-Wilk and Brown-Forsythe tests, respectively. Asterisks in the graphs indicate significance levels; ^*^P ≤ 0.05; ^**^P ≤ 0.01; ^***^P ≤ 0.001; n.s.: not significant.

### Experimental procedure

To address the issues encountered with conventionally used artificial two-dimensional growth surfaces, we manufactured plane as well as nano- and micrometer-patterned substrates with nominal pillar geometries of constant diameter (2 μm) and pitch (6 μm) but of varying height (130, 190, and 1,800 nm) using the elastomer types S-, H- and X-PDMS. Next we evaluated the characteristics with regard to wettability, roughness and applicability to cell-based approaches using HFF-1 cells (human foreskin fibroblasts) as a model system. A workflow of substrate production and characterization is shown in Figure 1.

## Results

We manufactured plane as well as nano- and micrometer-patterned substrates with nominal pillar geometries of constant diameter and pitch but of varying height using the elastomer types S-, H- and X-PDMS. First, the mechanical properties of the substrates were evaluated ny nano-indentation as described in the Methods section. According to the obtained mean values (±SD, *N* = 6) (2.5 ± 0.5 MPa, 7.0 ± 0.5 MPa, and 16.8 ± 1.3 MPa for S-, H-, and X-PDMS) the Young's modulus values are slightly smaller than reported in the literature (Verschuuren, 2010). In a next step we evaluated the characteristics with regard to wettability, roughness and applicability to cell-based approaches using HFF-1 cells (human foreskin fibroblasts) as a model system. A workflow of substrate production and characterization is shown in Figure 1.

### Characteristics of plane PDMS substrates

**Figure 2:**
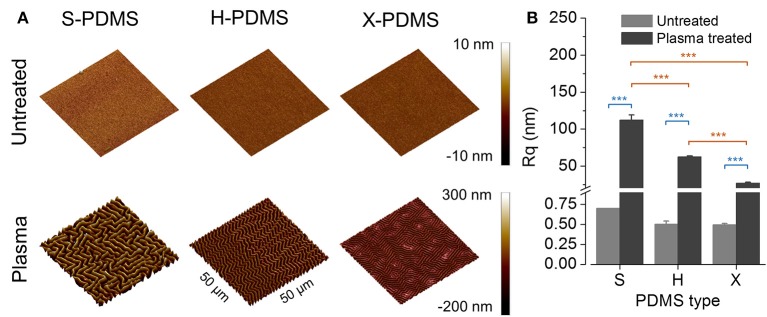
AFM analysis and root mean square roughness of plane PDMS substrates. **(A)** Three-dimensional reconstructions of AFM data recorded from PDMS substrates (50 × 50 μm^2^ scan area). **(B)** Root mean square roughness (R_q_) of untreated (gray bars) and plasma-treated (dark gray bars) PDMS substrates. Color coding of statistical analysis: within group “Plasma treated,” orange; between groups “Plasma treated” and “Untreated,” blue.

In a first step toward evaluating the characteristics of the manufactured PDMS growth substrates, we measured the roughness of the PDMS foils using atomic force microscopy as described in the methods section. Three-dimensional representations of the measured substrates are shown in Figure 2A. Figure 2B shows R_q_, i.e., the root mean square average of the roughness profile ordinates (mean ± SEM), of plane untreated (gray bars) and plasma-treated (dark gray bars) PDMS. R_q_ mean values and standard errors are included in Table S1 in the Supplements. These data demonstrate that the type of PDMS has only minor effect on the roughness when untreated. In contrast, S-, H-, and X-PDMS exhibit strongly different R_q_ values when treated with plasma. As the stiffness of PDMS increases in the sequence S-H-X, these results imply that the sensitivity to plasma treatment decreases along with increasing stiffness of the material.

**Figure 3:**
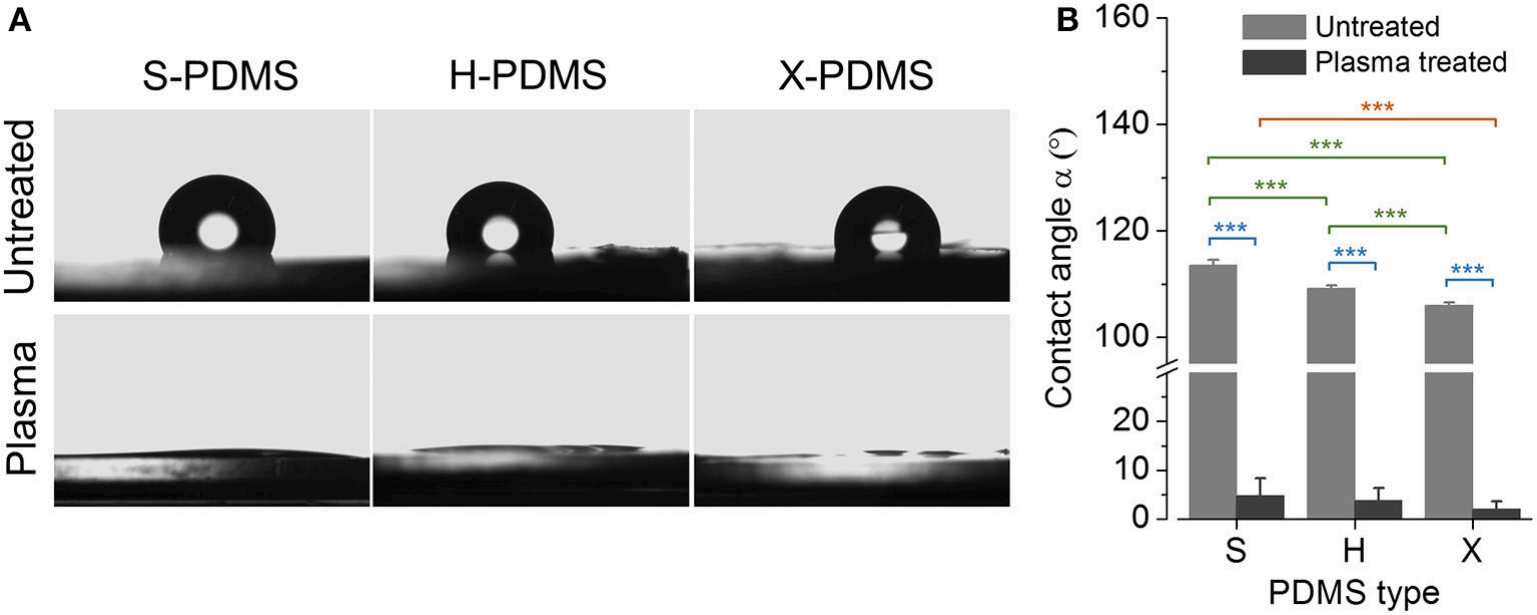
Water contact angle analysis of plane PDMS substrates. **(A)** Sample images of water droplets on untreated and plasma treated S-, H-, and X-PDMS surfaces acquired during quantification of the water contact angle. **(B)** Mean contact angle for untreated (gray bars) and plasma-treated (dark gray bars) PDMS. Color coding of statistical analysis: within group “Plasma treated,” orange; within group “Untreated,” green; between groups “Plasma treated” and “Untreated,” blue.

In the next step, we quantified the contact angle (α in °) of plane and untreated as well as plasma-treated S-, H-, and X-PDMS surfaces as a measure of wettability and suitability to cell-based approaches. Sample images of water droplets on untreated (upper images) and plasma treated (lower images) PDMS surfaces are shown in Figure 3A. The histogram in Figure 3B shows mean contact angles (±SEM) for plane untreated (gray bars) and plasma-treated (dark gray bars) PDMS, clearly indicating a significant difference in wettability between untreated and plasma treated elastomer but only minor differences between the individual PDMS types. Mean contact angles and standard errors are included in Table S2 in the Supplements.

**Figure 4:**
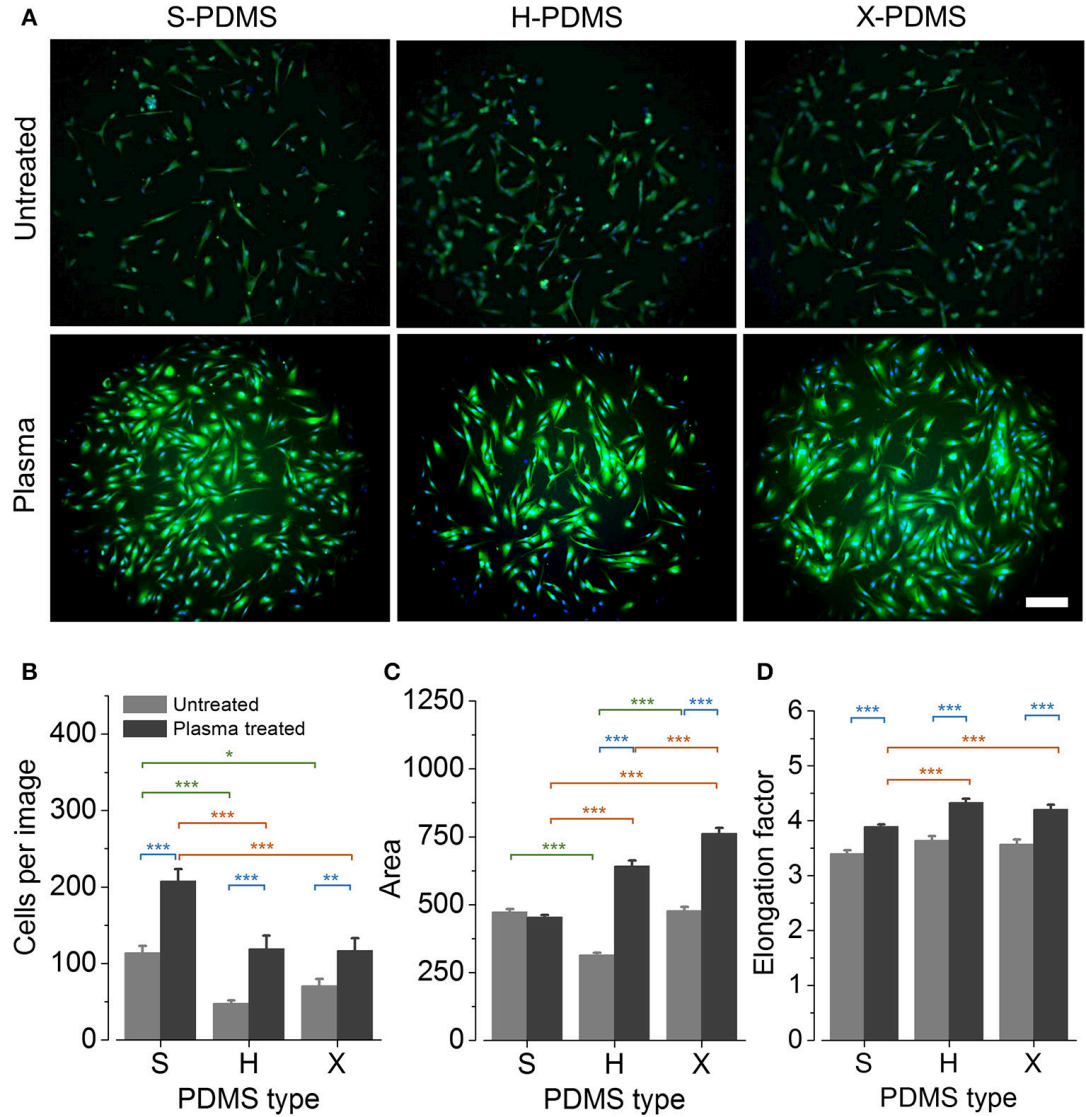
Viability and morphology of HFF-1 cells cultured on plane PDMS substrates. **(A)** Overlay images of Calcein (green) and Hoechst 33342 (blue) stained cells recorded with a 10x objective. Scale bar: 200 μm. **(B–D)** Mean cell number, cell area and elongation factor of cells quantified in images of HFF-1 cells cultured on the different PDMS types. *P ≤ 0.05; **P ≤ 0.01; ***P ≤ 0.001. Color coding of statistical analysis: within group “Plasma treated,” orange; within group “Untreated,” green; between groups “Plasma treated” and “Untreated,” blue.

In order to evaluate whether the quantified differences in the wettability of untreated and plasma treated PDMS substrates as well as the observed distinct roughness values for S-, H-, and X-PDMS are also reflected by cell-based data, we conducted a case study using HFF-1 cells as a model system. To this end, HFF-1 cells were cultured on PDMS substrates at defined density, stained with the fluorescent indicators Calcein-AM and Hoechst 33342 and were imaged with a high-content microscope as described in the Methods section. Figure 4A shows exemplary overlay images of Calcein (green) and Hoechst 33342 (blue) stained cells cultured on plane untreated and treated S-, H-, and X-PDMS, recorded with a 10x objective. The suitability of the PDMS substrates to cell-based approaches was assessed by analysing the number of cells, cell area and the elongation of cells, in fluorescence images, as measures of viability and adhesion to the growth surfaces. A low number of cells, a small cellular area as well as a round cellular morphology typically indicate cell death. Healthy and viable cells are usually characterized by a comparatively higher cell number, larger cell area and an elongated morphology (Galluzzi et al., 2007; Wei et al., 2007; Barnhart et al., 2011; Gilbert et al., 2011, 2016; Stanton et al., 2014; Dakhil et al., 2016; Gilbert and Boutros, 2016; Kuenzel et al., 2016; Schneidereit et al., 2016). Figures 4B–D show mean values (±SEM) of the cell number, the cell area and the mean elongation factor, respectively, for plane untreated (gray bars) and plasma treated (dark gray bars) PDMS. Mean values and standard errors of cell-derived data are included in Tables S3–S5 in the Supplements. For untreated samples H-PDMS shows smallest cell numbers and area whereas S- and X-PDMS show comparable cell area and significantly higher cell numbers. Regarding the elongation factor, all PDMS types show similar values. These data demonstrate the suitability of plasma-treated but not untreated PDMS foils to culturing HFF-1 cells in particular and to cell-based approaches in general. However, more importantly, our results demonstrate for the first time, that growth substrates fabricated from X-PDMS are as suitable for cell-based approaches as previously characterized S- and H-PDMS surfaces.

### Characteristics of patterned PDMS substrates

Plasma treatment is well established and reliable. However, it is also a non-permanent procedure if the substrates cannot be stored in appropriate storage media (Scharin et al., 2014) and the resulting topography is of somewhat random nature, rather than a controlled process of topographic design. Thus, topographies generated using plasma treatment are unpredictable and highly heterogeneous and for this reason unsuitable for systematically studying the influence of micro- or nanometer-scaled environmental structures on cellular viability or morphology.

In order to allow systematic studies addressing the interplay between e.g., micro or nanometer-sized environmental topographies and cells *in vitro* or *in vivo*, we have fabricated three different Si master wafers providing nano- or micrometer-structured hole topographies. Using the three elastomer types S-, H- and X-PDMS and the above mentioned Si wafers, we have prepared growth substrates of each PDMS type with nominal pillar geometries of constant diameter (2 μm) and pitch (6 μm) but of varying height (130, 190, and 1,800 nm).

**Figure 5:**
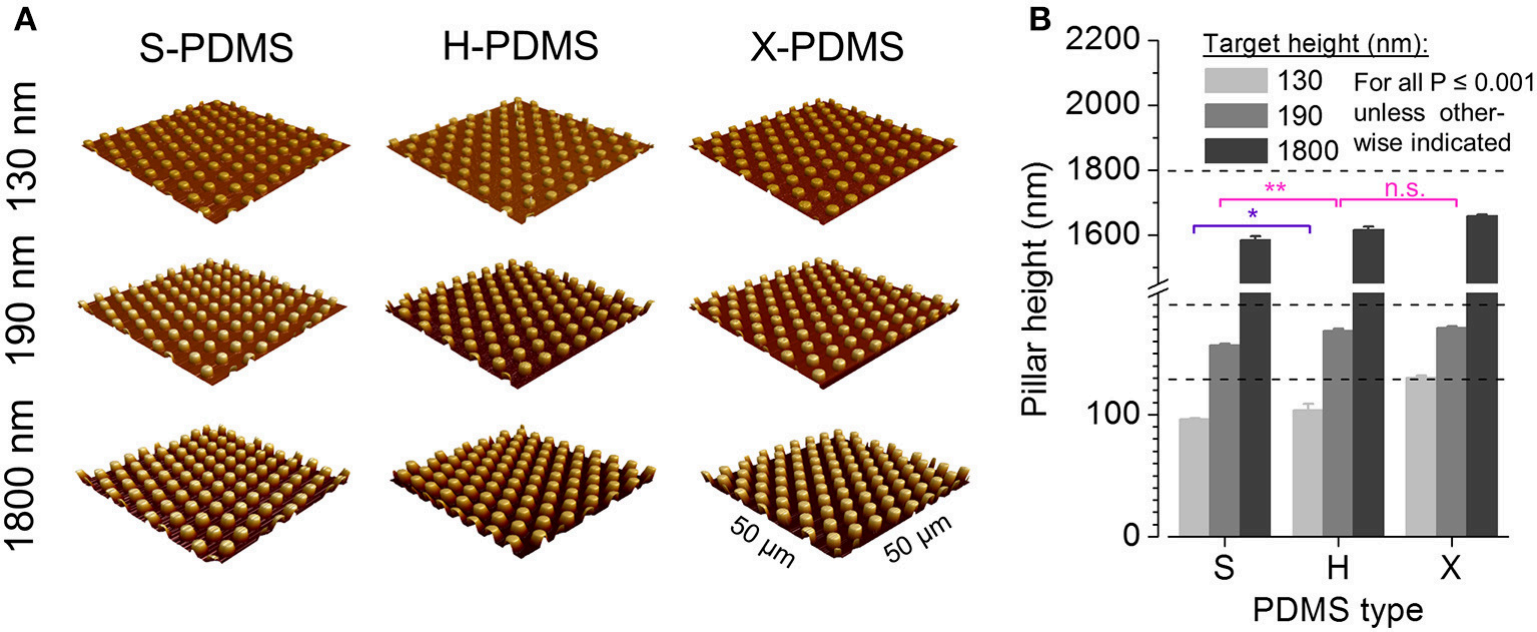
AFM analysis of structured PDMS substrates. **(A)** Three-dimensional reconstructions of fabricated pillar-structured PDMS substrates recorded by AFM. **(B)** Mean pillar height of plane S-, H-, and X-PDMS as measured by AFM. All data are significantly different at a significance level of *P* ≤ 0.001 as evaluated by two-way ANOVA unless otherwise indicated. Color coding of statistical analysis: within group “130 nm,” purple; within group “190 nm,” pink.

In an initial step prior to cell-based experimentation and in order to assess the accuracy of the molding process, we measured the pillar height of the manufactured PDMS substrates by atomic force microscopy as described in the Methods section. The corresponding scanning electron microscopy images are available in Figure S1 in the Supplements. Figure 5A shows representative three-dimensional reconstructions of the various PDMS foils. The mean pillar height (±SEM) measured for the different elastomer types S-, H- and X-PDMS for 130 (light gray), 190 (gray) and 1800 nm (dark gray) target height, respectively, is depicted in Figure 5B. The deviation from the target size (i.e., nominal hole depth of the master structures, see dotted lines in Figure 5B) increases in the sequence X-H-S. Additionally R_q_ increases in the sequence S-H-X and obviously with increasing pillar height (130–190–1,800 nm) as shown in Table 1. The mean pillar height and standard errors are included in Table S6 in the Supplements. The aforementioned results are somewhat expected as stiff material has previously been shown to be better suited for replicating nano- and micrometer-scaled structures compared to soft material (Verschuuren, 2010). Thus, the sequence of the molding accuracy simply follows the sequence of increasing Young's modulus of the different PDMS types.

**Table 1:** Root mean square roughness R_q_ of structured PDMS substrates in nm.

**Nom. height**	**S-PDMS**	**H-PDMS**	**X-PDMS**
130 nm	34.1 ± 0.3	35.8 ± 1.1	43.1 ± 0.7
190 nm	52.0 ± 0.2	62.4 ± 1.0	65.0 ± 1.9
1,800 nm	623.0 ± 0.8	649.4 ± 2.5	671.6 ± 5.7

**Figure 6:**
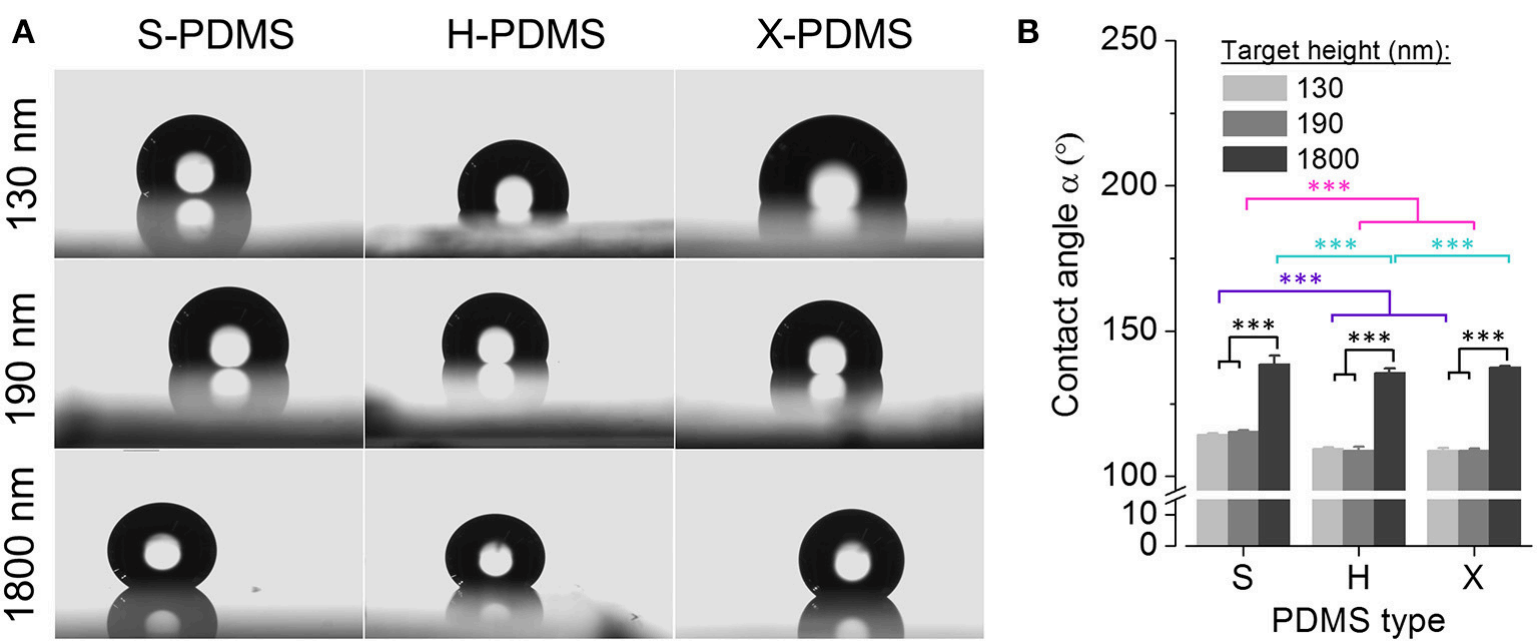
Water contact angle analysis of structured PDMS substrates. **(A)** Sample images of water droplets on PDMS surfaces for quantification of the water contact angle. **(B)** Mean water contact angle for S-, H-, and X-PDMS in structured configuration, i.e., 130 (light gray), 190 (gray) and 1,800 nm (dark gray) pillar target height, respectively. Color coding of statistical analysis: within group “130 nm,” purple; within group “190 nm,” pink; within group “1,800 nm,” turquoise; between groups “130 nm,” “190 nm,” and “1,800 nm” (either S-, H-, or X-PDMS), black.

In a next step, in order to assess the wettability of the fabricated PDMS substrates, we measured the WCA for the differently structured elastomer types. Sample images of water droplets on PDMS surfaces are shown in Figure 6A. Figure 6B shows mean contact angles (± SEM) for S-, H-, and X-PDMS in structured configuration, at 130 (light gray), 190 (gray), and 1,800 nm (dark gray) pillar target height, respectively. Mean values and standard errors are included in Table S7 in the Supplements. The measured WCAs consistently exceed 90°, confirming the Wenzel theory, predicting that a hydrophobic surface is rendered even more hydrophobic when the roughness (R_q_) is increased. The wettability of PDMS substrates decreases along with increasing height of the evaluated pillar-structured topography and R_q_, thus demonstrating that the wettability of PDMS can be altered by introducing nano- and micrometer-sized structures.

**Figure 7:**
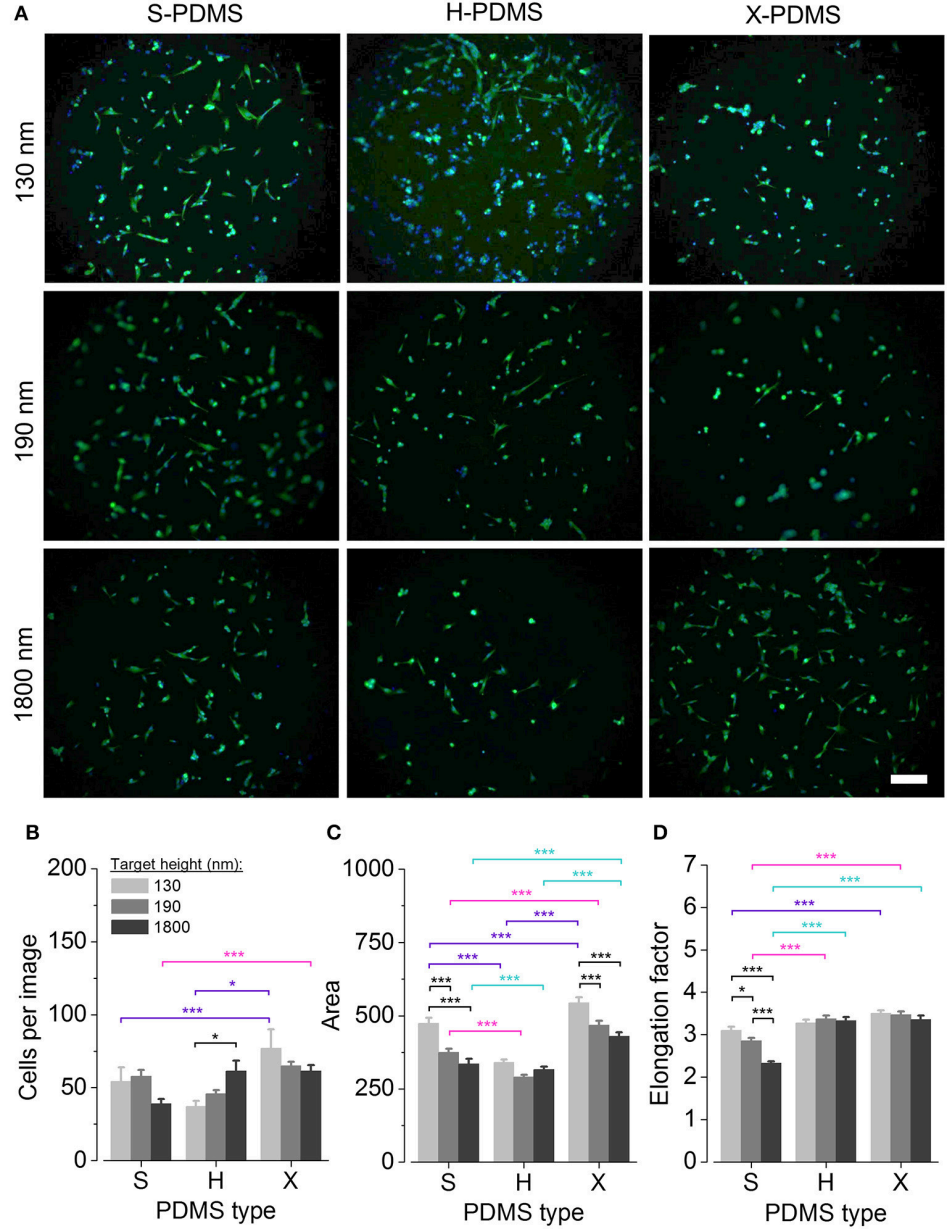
Viability and morphology of HFF-1 cells cultured on structured PDMS substrates. **(A)** Exemplary overlay images of Calcein (green) and Hoechst 33342 (blue) stained cells cultured on pillar-structured S-, H- and X-PDMS, recorded with a 10x objective. Scale bar: 200 μm. **(B–D)** Mean cell number, cell area and elongation factor, respectively, measured from cells cultured on structured S-, H-, and X-PDMS. *P ≤ 0.05; ***P ≤ 0.001; n.s. not significant. Color coding of statistical analysis: within group “130 nm,” purple; within group “190 nm,” pink; within group “1,800 nm,” turquoise; between groups “130 nm,” “190 nm,” and “1,800 nm” (either S-, H- or X-PDMS), black.

To evaluate whether the aforementioned wettability properties apply to cell-based data and to investigate that the fabricated pillar-structured PDMS foils are applicable for culturing cells, we quantified cell number, cell area and cellular elongation equally to the experiments with non-structured PDMS substrates (see Methods for details). Figure 7A shows exemplary overlay images of Calcein (green) and Hoechst 33342 (blue) stained cells cultured on pillar-structured S-, H-, and X-PDMS. Figures 7B–D show the cell number (mean ± SEM), the cell area (mean ± SEM) as well as the elongation factor for S-, H- and X-PDMS) in structured configuration, i.e., 130 (light gray), 190 (gray), and 1,800 nm (dark gray) pillar target height, respectively. Mean values and standard errors of cell-derived data are included in Tables S8–S10 in the Supplements. For S-PDMS, the cell area and the elongation but not the cell number significantly decreases along with increasing pillar height. Thus, most of the employed indicators reflect data obtained by wettability analysis. For H-PDMS, the cell number significantly increases between the short pillars (130 and 190 nm) and the long pillars. In turn, no significant pillar height-dependent change could be observed when looking at cell area and cell elongation. For X-PDMS, the cell number as well as the elongation factor indicates no significant differences between the various pillar heights, whereas the cell area significantly decreases along with increasing pillar height, hence reflecting data obtained by wettability analysis as also observed for S-PDMS. Despite the fact that the variation in pillar height is only inconsistently and partly contradictorily reflected by the employed indirect indicators of cell viability and adherence, structured X-PDMS elicits no significant difference compared to the previously described elastomer types S- and H-PDMS.

## Discussion

We have designed and fabricated plane as well as nano- and micrometer-scaled pillar-patterned growth substrates using the elastomer types S-, H-, and X-PDMS. In order to obtain an estimate regarding the applicability for cell-based approaches, we evaluated the characteristics of the generated surfaces with regard to wettability and roughness using WCA measurement and atomic force microscopy, respectively. We further assessed cell number and morphology as measures of cellular viability and adhesion by image cytometry and phenotypic profiling, respectively, using Calcein and Hoechst 33342 stained human foreskin fibroblasts as a model system.

### Characteristics of plane PDMS substrates

We measured the Young's modulus values of the as-prepared samples and our values slightly differ from results reported in literature (Verschuuren, 2010). A possible reason might be that in our work we used the component HMS-301 whereas HMS-501 was used in the reference work (Verschuuren, 2010) which cures faster than HMS-301. Therefore, HMS-501 could lead to higher crosslinking, which in turn might result in a slightly higher Young's modulus.

We show that the different PDMS types are differently sensitive to plasma treatment resulting in different and increasing R_q_ values in the sequence X-H-S PDMS, implying that the sensitivity to plasma treatment decreases along with increasing stiffness of the material. Following the Wenzel-Theory an increasing roughness should lead to increasing WCA for our samples. However, the very low WCA for all plasma treated surfaces suggest that the chemical modification or interaction of surface groups predominates, not the roughness of PDMS surfaces. Based on WCA measurements, for plane PDMS substrates, we confirm earlier research showing that hydrophobic PDMS substrates are turned into hydrophilic surfaces by plasma treatment—regardless of the PDMS type. WCAs below 90° have been reported to support cellular adhesion (Gittens et al., 2014; Rupp et al., 2014), hence, these data suggest that plasma treated S-, H-, and X-PDMS substrates are suitable to application with *in vitro* cultures.

Plasma treated samples show significantly higher values of the average cell number, the cell area and the median elongation factor for plasma-treated compared to untreated PDMS. This correlates with the low WCA values of all plasma-treated surfaces indicating good suitability to application with *in vitro* cultures. Only the area of cells, cultured on untreated and plasma treated S-PDMS indicates no significant difference. This phenomenon may be explained by a significantly higher (approx. doubled) number of cells on treated substrates vs. untreated (S-PDMS) surfaces and a resulting limitation of the cell area. This hypothesis is supported by the presence of fewer and larger cells observed on plasma treated H- and X-PDMS. The number of cells cultured on plasma treated S-PDMS substrate is significantly higher than for plasma treated X- and H-PDMS, indicating that the softest and roughest PDMS is suited best for application with cells. This observation compares well with data from the literature reporting an increasing rate in cell proliferation along with decreasing PDMS stiffness (Brown et al., 2005).

However, for plasma treated surfaces, the cell area increases in the sequence S-H-X, indicating increased cellular adherence in the same sequence (though cell area for plasma treated S-PDMS might be small due to limited overall area). Although these data seemingly oppose measurements implied by the cell number, these results are also reflected by the cellular elongation. Looking at the elongation factor, cells cultured on the softest and roughest surface elicited the smallest elongation factors, thus, the roundest morphotypes. A comparably smaller cell area and elongation factor would be expected for cells cultured at saturating confluency vs. cells maintained at lower confluency. Despite the fact that these observations have not been evaluated in greater detail, cells cultured on plasma treated S-PDMS display the highest number of cells quantified for all conditions, indicating high confluency and supporting the aforementioned hypothesis. The results indicate that the mechanical properties of PDMS materials and surface roughness after plasma treatment play an important role for cell adhesion and proliferation (Bartalena et al., 2012; Seo et al., 2013).

Our results obtained with plane PDMS substrates show that both, wettability and roughness contribute to the ability of cells to interact, i.e., adhere, to plane PDMS growth substrates and thus confirm earlier reports in the literature. Furthermore, despite a rather small variation in wettability along with varying roughness between different PDMS types as quantified by contact angle measurement and AFM, as well as partly contradictive results observed regarding cell-based data, there is clear evidence that alteration of roughness results in significant differences in the cells' ability to proliferate and adhere. In addition, these results confirm that S- and H-PDMS are suitable to cell-based approaches and introduce X-PDMS for *in vitro* applications.

### Characteristics of structured PDMS substrates

With regard to structured PDMS, we provide evidence from atomic force microscopy that X-PDMS is suited best for molding nanometer-scaled geometries and yields higher pattern accuracy compared to H- and S-PDMS. Using WCA measurements, we show that the wettability of PDMS substrates decreases along with increasing height of the evaluated pillar-structured topography and R_q_,. It is clearly visible, that for all PDMS types small pillars (130 and 190 nm height) only slightly change the contact angle compared to plane samples, whereas the contact angle significantly increases for the pillars with largest heights with considerably increased R_q_,. At small pillar heights it is assumed that the water droplet fills the space between the pillars. With the significantly increased pillar height up to 1,800 nm also a Cassie-Baxter state might occur where the droplet does not penetrate the spaces between the pillars. Despite increased R_q_ of H- and X-PDMS compared to S-PDMS and an expected relatively higher WCA, all patterned S-PDMS surfaces exhibit lower wettability. Furthermore, it is striking, that H- and X-PDMS surfaces for plane and all patterned surfaces show higher wettability compared to S-PDMS. H- and X-PDMS exhibit a stronger chemical interaction with water, probably due to the existence of relatively short cross-linkers on the surfaces of H- and X-PDMS (Choi and Rogers, 2003).

Our cell-based experiments indicate that cellular adhesion on structured growth surfaces cannot exclusively be explained by the surfaces' wettability. Comparing the results for structured and unstructured samples (as shown in Figures 4, 7), no clear trend can be observed. Unstructured S-PDMS showed the highest cell number from all samples but, e.g., for X-PDMS structured and unstructured samples show comparable cell numbers. Also, cell area and elongation factor for patterned surfaces indicate good health state and adhesion of the cells despite the strong hydrophobicity of the structured surfaces (see Figure 6). As e.g., unpatterned, untreated S-PDMS is well known for its bio- and cytocompatibility, this in turn shows that structured PDMS samples could also be considered for biomedical applications with the additional advantage of enabling ECM-like growth substrates.

The results derived from structured PDMS substrates demonstrate that the wettability of PDMS in general can be altered by introducing nano- and micrometer-sized pillar structures. The measured wettability properties are only reflected to a certain extent by cell-based data, revealing expected but also contradictive results for the various elastomer types and indicate that cellular adhesion on growth surfaces cannot exclusively be explained by the surfaces' wettability. In order to gain a deeper insight into the mechanisms of the observed phenomena, further experiments, assessing, e.g., the expression and distribution of focal adhesion or cytoskeletal proteins enabling cell-substrate-interactions are required. However, our cell-based data obtained with X-PDMS are overall very promising as this material may be suitable for the generation of structure sizes and topographies in the low nanometer size-range for production of biomedical devices with improved, e.g., ECM-like characteristics. We hypothesize that nanometer-sized structuring of X-PDMS may serve as a powerful alternative to plasma treatment for supporting cellular adhesion and viability.

Most importantly, we show for the first time that structured X-PDMS elicits no significant difference with respect to cytocompatibility and adhesive properties compared to the previously described elastomer types S- and H-PDMS, thus promoting the applicability of elastomer growth substrates for the development of biomedical devices with improved properties.

It is important to mention, that the structures fabricated for the presented study are large compared to native ECM-associated structures, such as collagen filaments, leaving great potential for further experimentation with nanometer-sized growth substrates, using X-PDMS. Indeed, AFM data obtained from plasma-treated and “attachment-friendly” PDMS foils indicate that the roughened substrates elicit structure heights in the lower nanometer size range. Hence, nanometer-sized structuring of X-PDMS may serve as a powerful alternative to plasma treatment for supporting cellular adhesion and viability.

As our data also indicate slightly decreased cellular viability and adherence for the highest pillar structures, such topographies may be useful as particularly “attachment-unfriendly” areas in lab-on-a-chip devices representing compartments with endothelial or even bactericidal properties (Karahaliloglu et al., 2015; Serrano et al., 2015). Altogether, this work contributes to furthering the applicability and availability of plane and structured PDMS.

## Author contributions

MS-M, MR, TD, OF, LF, and DFG conceived the project and designed experiments. MS-M and AH conducted experiments. MS-M, AH, and DFG analyzed and displayed the data. MS-M and DFG wrote the paper.

### Conflict of interest statement

The authors declare that the research was conducted in the absence of any commercial or financial relationships that could be construed as a potential conflict of interest.

## References

[B1] Ambrosia M. S. Ha M. Y. Balachandar S. (2013). The effect of pillar surface fraction and pillar height on contact angles using molecular dynamics. Appl. Surf. Sci. 282, 211–216. 10.1016/j.apsusc.2013.05.104

[B2] Barnhart E. L. Lee K. C. Keren K. Mogilner A. Theriot J. A. (2011). An adhesion-dependent switch between mechanisms that determine motile cell shape. PLoS Biol. 9:e1001059. 10.1371/journal.pbio.1001059 21559321 PMC3086868

[B3] Bartalena G. Loosli Y. Zambelli T. Snedeker J. G. (2012). Biomaterial surface modifications can dominate cell-substrate mechanics: the impact of PDMS plasma treatment on a quantitative assay of cell stiffness. Soft Matter 8, 673–681. 10.1039/C1SM06250F

[B4] Bettinger C. J. Langer R. Borenstein J. T. (2009). Engineering substrate topography at the micro- and nanoscale to control cell function. Angew. Chem. Int. Ed. Engl. 48, 5406–5415. 10.1002/anie.200805179 19492373 PMC2834566

[B5] Bhushan B. Jung Y. C. (2006). Micro- and nanoscale characterization of hydrophobic and hydrophilic leaf surfaces. Nanotechnology 17, 2758–2772. 10.1088/0957-4484/17/11/008

[B6] Bhushan B. Nosonovsky M. Jung C. Y. (2008). Lotus effect: roughness-induced superhydrophobic surfaces, in Nanotribology and Nanomechanics: An Introduction, ed Bhushan B. (Berlin; Heidelberg: Springer), 995–1072.

[B7] Bodas D. Rauch J.-Y. Khan-Malek C. (2008). Surface modification and aging studies of addition-curing silicone rubbers by oxygen plasma. Eur. Polym. J. 44, 2130–2139. 10.1016/j.eurpolymj.2008.04.012

[B8] Braut-Boucher F. Pichon J. Rat P. Adolphe M. Aubery M. Font J. (1995). A non-isotopic, highly sensitive, fluorimetric, cell-cell adhesion microplate assay using calcein AM-labeled lymphocytes. J. Immunol. Methods 178, 41–51. 10.1016/0022-1759(94)00239-S 7829864

[B9] Brown X. Q. Ookawa K. Wong J. Y. (2005). Evaluation of polydimethylsiloxane scaffolds with physiologically-relevant elastic moduli: interplay of substrate mechanics and surface chemistry effects on vascular smooth muscle cell response. Biomaterials 26, 3123–3129. 10.1016/j.biomaterials.2004.08.009 15603807

[B10] Chambers K. F. Mosaad E. M. Russell P. J. Clements J. A. Doran M. R. (2014). 3D Cultures of prostate cancer cells cultured in a novel high-throughput culture platform are more resistant to chemotherapeutics compared to cells cultured in monolayer. PLoS ONE 9:e111029. 10.1371/journal.pone.0111029 25380249 PMC4224379

[B11] Choi K. M. Rogers J. A. (2003). A photocurable poly(dimethylsiloxane) chemistry designed for soft lithographic molding and printing in the nanometer regime. J. Am. Chem. Soc. 125, 4060–4061. 10.1021/ja029973k 12670222

[B12] Cox J. D. Curry M. S. Skirboll S. K. Gourley P. L. Sasaki D. Y. (2002). Surface passivation of a microfluidic device to glial cell adhesion: a comparison of hydrophobic and hydrophilic SAM coatings. Biomaterials 23, 929–935. 10.1016/S0142-9612(01)00205-8 11771713

[B13] Cukierman E. Pankov R. Stevens D. R. Yamada K. M. (2001). Taking cell-matrix adhesions to the third dimension. Science 294, 1708–1712. 10.1126/science.1064829 11721053

[B14] Dakhil H. Gilbert D. F. Malhotra D. Limmer A. Engelhardt H. Amtmann A. (2016). Measuring average rheological quantities of cell monolayers in the linear viscoelastic regime. Rheologica Acta 55, 527–536. 10.1007/s00397-016-0936-5

[B15] Discher D. E. Janmey P. Wang Y.-L. (2005). Tissue cells feel and respond to the stiffness of their substrate. Science 310, 1139–1143. 10.1126/science.1116995 16293750

[B16] Dowling D. P. Miller I. S. Ardhaoui M. Gallagher W. M. (2011). Effect of surface wettability and topography on the adhesion of osteosarcoma cells on plasma-modified polystyrene. J. Biomater. Appl. 26, 327–347. 10.1177/0885328210372148 20566655

[B17] Doyle A. D. Petrie R. J. Kutys M. L. Yamada K. M. (2013). Dimensions in cell migration. Curr. Opin. Cell Biol. 25, 642–649. 10.1016/j.ceb.2013.06.004 23850350 PMC3758466

[B18] Fritz J. L. Owen M. J. (1995). Hydrophobic recovery of plasma-treated polydimethylsiloxane. J. Adhes. 54, 33–45. 10.1080/00218469508014379

[B19] Galluzzi L. Maiuri M. C. Vitale I. Zischka H. Castedo M. Zitvogel L.. (2007). Cell death modalities: classification and pathophysiological implications. Cell Death Differ. 14, 1237–1243. 10.1038/sj.cdd.4402148 17431418

[B20] Ghosh K. Ingber D. E. (2007). Micromechanical control of cell and tissue development: implications for tissue engineering. Adv. Drug Deliv. Rev. 59, 1306–1318. 10.1016/j.addr.2007.08.014 17920155

[B21] Gilbert D. F. Boutros M. (2016). A protocol for a high-throughput multiplex cell viability assay. Methods Mol. Biol. 1470, 75–84. 10.1007/978-1-4939-6337-9_6 27581285

[B22] Gilbert D. F. Erdmann G. Zhang X. Fritzsche A. Demir K. Jaedicke A.. (2011). A novel multiplex cell viability assay for high-throughput RNAi screening. PLoS ONE 6:e28338. 10.1371/journal.pone.0028338 22162763 PMC3230607

[B23] Gilbert D. F. Meinhof T. Pepperkok R. Runz H. (2009). DetecTiff: a novel image analysis routine for high-content screening microscopy. J. Biomol. Screen. 14, 944–955. 10.1177/1087057109339523 19641223

[B24] Gilbert D. F. Stebbing M. J. Kuenzel K. Murphy R. M. Zacharewicz E. Buttgereit A.. (2016). Store-operated Ca^2+^ entry (SOCE) and purinergic receptor-mediated Ca^2+^ homeostasis in murine bv2 microglia cells: early cellular responses to ATP-mediated microglia activation. Front. Mol. Neurosci. 9:111. 10.3389/fnmol.2016.00111 27840602 PMC5083710

[B25] Gilmour A. D. Woolley A. J. Poole-Warren L. A. Thomson C. E. Green R. A. (2016). A critical review of cell culture strategies for modelling intracortical brain implant material reactions. Biomaterials 91, 23–43. 10.1016/j.biomaterials.2016.03.011 26994876

[B26] Gittens R. A. Scheideler L. Rupp F. Hyzy S. L. Geis-Gerstorfer J. Schwartz Z.. (2014). A review on the wettability of dental implant surfaces II: biological and clinical aspects. Acta Biomater. 10, 2907–2918. 10.1016/j.actbio.2014.03.032 24709541 PMC4103435

[B27] Gomathi N. Sureshkumar A. Neogi S. (2008). RF plasma-treated polymers for biomedical applications. Curr. Sci. 94, 1478–1486. Available online at: http://www.jstor.org/stable/24100504

[B28] Green J. A. Yamada K. M. (2007). Three-dimensional microenvironments modulate fibroblast signaling responses. Adv. Drug Deliv. Rev. 59, 1293–1298. 10.1016/j.addr.2007.08.005 17825946 PMC2140276

[B29] Guan Z. Y. Wu C. Y. Li Y. J. Chen H. Y. (2015). Switching the biointerface of displaceable poly-p-xylylene coatings. ACS Appl. Mater. Interfaces 7, 14431–14438. 10.1021/acsami.5b03286 26084053

[B30] Halldorsson S. Lucumi E. Gómez-Sjöberg R. Fleming R. M. T. (2015). Advantages and challenges of microfluidic cell culture in polydimethylsiloxane devices. Biosens. Bioelectron. 63, 218–231. 10.1016/j.bios.2014.07.029 25105943

[B31] Hillborg H. Gedde U. W. (1998). Hydrophobicity recovery of polydimethylsiloxane after exposure to corona discharges. Polymer 39, 1991–1998. 10.1016/S0032-3861(97)00484-9

[B32] Hwang S. Y. Kwon K. W. Jang K.-J. Park M. C. Lee J. S. Suh K. Y. (2010). Adhesion assays of endothelial cells on nanopatterned surfaces within a microfluidic channel. Anal. Chem. 82, 3016–3022. 10.1021/ac100107z 20218573

[B33] Jellali R. Duval J. L. Leclerc E. (2016). Analysis of the biocompatibility of perfluoropolyether dimethacrylate network using an organotypic method. Mater. Sci. Eng. C Mater. Biol. Appl. 65, 295–302. 10.1016/j.msec.2016.04.057 27157755

[B34] Karahaliloglu Z. Ercan B. Taylor E. N. Chung S. Denkbaş E. B. Webster T. J. (2015). Antibacterial nanostructured polyhydroxybutyrate membranes for guided bone regeneration. J. Biomed. Nanotechnol. 11, 2253–2263. 10.1166/jbn.2015.2106 26510318

[B35] Keshavarz M. Tan B. Venkatakrishnan K. (2016). Functionalized stress component onto bio-template as a pathway of cytocompatibility. Sci. Rep. 6:35425. 10.1038/srep35425 27759054 PMC5069693

[B36] Kuenzel K. Friedrich O. Gilbert D. F. (2016). A recombinant human pluripotent stem cell line stably expressing halide-sensitive YFP-I152L for GABAAR and GlyR-targeted high-throughput drug screening and toxicity testing. Front. Mol. Neurosci. 9:51. 10.3389/fnmol.2016.00051 27445687 PMC4923258

[B37] Larsson R. Nygren P. (1989). A rapid fluorometric method for semiautomated determination of cytotoxicity and cellular proliferation of human tumor cell lines in microculture. Anticancer Res. 9, 1111–1119. 2817793

[B38] Li Y. Kilian K. A. (2015). Bridging the gap: from 2D cell culture to 3D microengineered extracellular matrices. Adv. Healthc. Mater. 4, 2780–2796. 10.1002/adhm.201500427 26592366 PMC4780579

[B39] Martínez E. Engel E. Planell J. A. Samitier J. (2009). Effects of artificial micro- and nano-structured surfaces on cell behaviour. Ann. Anat. Anat. Anz. 191, 126–135. 10.1016/j.aanat.2008.05.006 18692370

[B40] Menzner A. K. Abolpour Mofrad S. Friedrich O. Gilbert D. F. (2015). Towards *in vitro* DT/DNT testing: assaying chemical susceptibility in early differentiating NT2 cells. Toxicology 338, 69–76. 10.1016/j.tox.2015.10.007 26498558

[B41] Millet L. J. Gillette M. U. (2012). Over a century of neuron culture: from the hanging drop to microfluidic devices. Yale J. Biol. Med. 85, 501–521. 23239951 PMC3516892

[B42] Moyen E. Hama A. Ismailova E. Assaud L. Malliaras G. Hanbücken M.. (2016). Nanostructured conducting polymers for stiffness controlled cell adhesion. Nanotechnology 27:074001. 10.1088/0957-4484/27/7/074001 26790487

[B43] Owen M. J. Smith P. J. (1994). Plasma treatment of polydimethylsiloxane. J. Adhes. Sci. Technol. 8, 1063–1075. 10.1163/156856194X00942

[B44] Peterson S. L. McDonald A. Gourley P. L. Sasaki D. Y. (2005). Poly(dimethylsiloxane) thin films as biocompatible coatings for microfluidic devices: cell culture and flow studies with glial cells. J. Biomed. Mater. Res. A 72, 10–18. 10.1002/jbm.a.30166 15534867

[B45] Quéré D. (2008). Wetting and roughness. Annu. Rev. Mater. Res. 38, 71–99. 10.1146/annurev.matsci.38.060407.132434

[B46] Ranella A. Barberoglou M. Bakogianni S. Fotakis C. Stratakis E. (2010). Tuning cell adhesion by controlling the roughness and wettability of 3D micro/nano silicon structures. Acta Biomater. 6, 2711–2720. 10.1016/j.actbio.2010.01.016 20080216

[B47] Rupp F. Gittens R. A. Scheideler L. Marmur A. Boyan B. D. Schwartz Z.. (2014). A review on the wettability of dental implant surfaces I: theoretical and experimental aspects. Acta Biomater. 10, 2894–2906. 10.1016/j.actbio.2014.02.040 24590162 PMC4041806

[B48] Sánchez-Romero N. Schophuizen C. M. Giménez I. Masereeuw R. (2016). *In vitro* systems to study nephropharmacology: 2D versus 3D models. Eur. J. Pharmacol. 10.1016/j.ejphar.2016.07.010 27395797

[B49] Scharin M. Rommel M. Dirnecker T. Marhenke J. Herrmann B. Rumler M. (2014). Bioactivation of plane and patterned PDMS thin films by wettability engineering. Bionanoscience 4, 251–262. 10.1007/s12668-014-0145-6

[B50] Schmitt H. Duempelmann P. Fader R. Rommel M. Bauer A. J. Frey L. (2012). Life time evaluation of PDMS stamps for UV-enhanced substrate conformal imprint lithography. Microelectron. Eng. 98, 275–278. 10.1016/j.mee.2012.04.032

[B51] Schneidereit D. Kraus L. Meier J. C. Friedrich O. Gilbert D. F. (2016). Step-by-step guide to building an inexpensive 3D printed motorized positioning stage for automated high-content screening microscopy. Biosens. Bioelectron. 92, 472–481. 10.1016/j.bios.2016.10.078 27840039

[B52] Seo J. H. Sakai K. Yui N. (2013). Adsorption state of fibronectin on poly(dimethylsiloxane) surfaces with varied stiffness can dominate adhesion density of fibroblasts. Acta Biomater. 9, 5493–5501. 10.1016/j.actbio.2012.10.015 23088883

[B53] Serrano C. García-Fernández L. Fernández-Blázquez J. P. Barbeck M. Ghanaati S. Unger R.. (2015). Nanostructured medical sutures with antibacterial properties. Biomaterials 52, 291–300. 10.1016/j.biomaterials.2015.02.039 25818435

[B54] Song F. Ren D. (2014). Stiffness of cross-linked poly(Dimethylsiloxane) affects bacterial adhesion and antibiotic susceptibility of attached cells. Langmuir 30, 10354–10362. 10.1021/la502029f 25117376

[B55] Stanton M. M. Rankenberg J. M. Park B. W. McGimpsey W. G. Malcuit C. Lambert C. R. (2014). Cell behavior on surface modified polydimethylsiloxane (PDMS). Macromol. Biosci. 14, 953–964. 10.1002/mabi.201300504 24599684

[B56] van Kooten T. G. Whitesides J. F. von Recum A. (1998). Influence of silicone (PDMS) surface texture on human skin fibroblast proliferation as determined by cell cycle analysis. J. Biomed. Mater. Res. 43, 1–14. 9509339 10.1002/(sici)1097-4636(199821)43:1<1::aid-jbm1>3.0.co;2-t

[B57] Verschuuren M. A. (2010). Substrate Conformal Imprint Lithography for Nanophotonics. Utrecht: Utrecht University.

[B58] Wei J. Yoshinari M. Takemoto S. Hattori M. Kawada E. Liu B.. (2007). Adhesion of mouse fibroblasts on hexamethyldisiloxane surfaces with wide range of wettability. J. Biomed. Mater. Res. B Appl. Biomater. 81, 66–75. 10.1002/jbm.b.30638 16924616

[B59] Wenzel R. N. (1936). Resistance of solid surfaces to wetting by water. Ind. Eng. Chem. 28, 988–994. 10.1021/ie50320a024

[B60] Wrzesinski K. Fey S. J. (2015). From 2D to 3D–a new dimension for modelling the effect of natural products on human tissue. Curr. Pharm. Des. 21, 5605–5616. 10.2174/1381612821666151002114227 26429710

[B61] Wu T. H. Li C. H. Tang M. J. Liang J. I. Chen C. H. Yeh M. L. (2013). Migration speed and directionality switch of normal epithelial cells after TGF-beta1-induced EMT (tEMT) on micro-structured polydimethylsiloxane (PDMS) substrates with variations in stiffness and topographic patterning. Cell Commun. Adhes. 20, 115–126. 10.3109/15419061.2013.833194 24053415

[B62] Yamamoto H. Demura T. Morita M. Kono S. Sekine K. Shinada T.. (2014). *In situ* modification of cell-culture scaffolds by photocatalytic decomposition of organosilane monolayers. Biofabrication 6:035021. 10.1088/1758-5082/6/3/035021 25100800

[B63] Yim E. K. Reano R. M. Pang S. W. Yee A. F. Chen C. S. Leong K. W. (2005). Nanopattern-induced changes in morphology and motility of smooth muscle cells. Biomaterials 26, 5405–5413. 10.1016/j.biomaterials.2005.01.058 15814139 PMC2376810

[B64] Young M. Reed K. R. (2016). Organoids as a Model for Colorectal Cancer. Curr. Colorectal Cancer Rep. 12, 281–287. 10.1007/s11888-016-0335-4 27656116 PMC5016547

[B65] Zhou J. Khodakov D. A. Ellis A. V. Voelcker N. H. (2012). Surface modification for PDMS-based microfluidic devices. Electrophoresis 33, 89–104. 10.1002/elps.201100482 22128067

[B66] Zilio C. Sola L. Damin F. Faggioni L. Chiari M. (2014). Universal hydrophilic coating of thermoplastic polymers currently used in microfluidics. Biomed. Microdevices 16, 107–114. 10.1007/s10544-013-9810-8 24037663

