# Supplementary material for: Nano- and Micro-Patterned S-, H-, and X-PDMS for Cell-Based Applications: Comparison of Wettability, Roughness, and Cell-Derived Parameters
Source: Front Bioeng Biotechnol. 2018 May 1;6:51. doi: 10.3389/fbioe.2018.00051 (PMC5938557; doi:10.3389/fbioe.2018.00051)
Supplement: Supplementary file 1 [file Data_Sheet_1.pdf]

Nano- and micro-patterned S-, H- and X-PDMS for cell-based applications: Comparison of wettability, roughness and cell-derived parameters

Marina Scharin-Mehlmann[a], Aaron Haering[a,b], Mathias Rommel[c], Tobias Dirnecker[a], Oliver Friedrich[b,d], Lothar Frey[a,c,d] and Daniel F. Gilbert[b,d]

[a] *Chair of Electron Devices, Friedrich-Alexander-Universität Erlangen-Nürnberg, Erlangen 91058, Germany.*

[b] *Institute of Medical Biotechnology, Friedrich-Alexander-Universität Erlangen-Nürnberg, Erlangen 91052, Germany.*

[c] *Fraunhofer Institute for Integrated Systems and Device Technology (IISB), 91058 Erlangen, Germany.*

[d] *Erlangen Graduate School in Advanced Optical Technologies (SAOT), Paul-Gordan-Straße 6, 91052 Erlangen, Germany*

Supplementary information

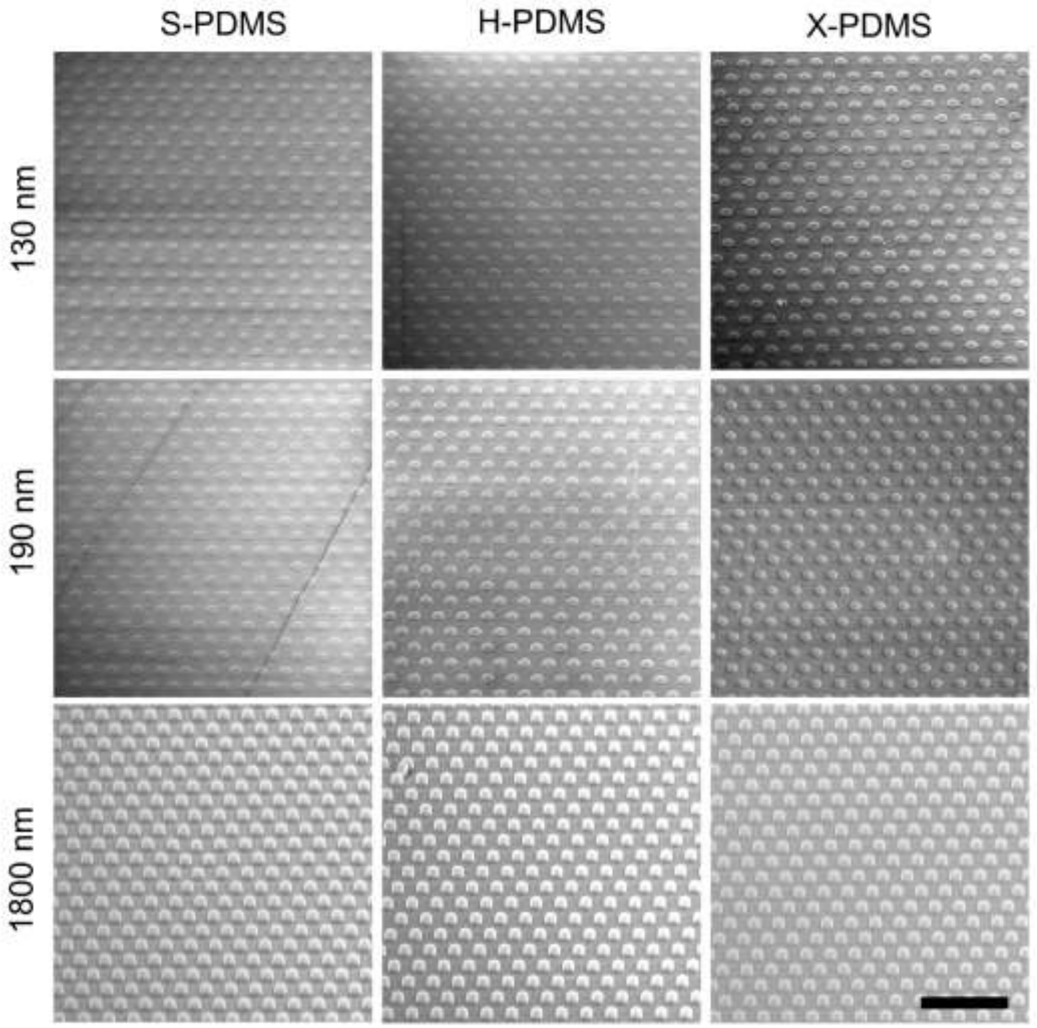

Figure S1. Scanning electron microscopy images of S-, H- and X-PDMS substrates. The displayed pillars are of constant diameter (2 µm) and pitch (6 µm) but of varying height as indicated. Scar bar: 20 µm.

Table S1. R_q mean values and standard errors for plane PDMS samples.

PDMS type	Untreated			Plasma treated		
	Mean	S.E.M	N	Mean	S.E.M	N
**S**	0.75	0.58	5	112	6	5
**H**	0.5	0.03	5	62	1	5
**X**	0.5	0.01	5	27	1	5

Table S2. Mean contact angles and standard errors.

PDMS type	Untreated			Plasma treated		
	Mean	S.E.M	N	Mean	S.E.M	N
**S**	113.5	1	10	4.7	3.5	10
**H**	109.1	0.5	10	3.6	2.6	10
**X**	105.9	0.6	10	2	1.5	10

Table S3. Mean cell number and standard error.

PDMS type	Untreated			Plasma treated		
	Mean	S.E.M	N	Mean	S.E.M	N
**S**	114.1	9	10	208	15.4	10
**H**	48.1	3.5	12	119.1	17.1	9
**X**	71.1	8.5	10	117	15.8	10

Table S4. Mean cell area and standard error.

PDMS type	Untreated			Plasma treated		
	Mean	S.E.M	N	Mean	S.E.M	N
**S**	472	11	1141	452	8	2080
**H**	314	8	577	642	20	1072
**X**	477	14	711	762	19	1170

Table S5. Mean cellular elongation factor and standard error.

PDMS type	Untreated			Plasma treated		
	Mean	S.E.M	N	Mean	S.E.M	N
**S**	3.4	0.1	1141	3.8	0.1	2080
**H**	3.6	0.1	577	4.3	0.1	1072
**X**	3.5	0.1	711	4.2	0.1	1170

Table S6. Mean pillar height and standard error.

PDMS type	130 nm				190 nm				1800 nm			
	Mean	S.E.M	% Deviation	N	Mean	S.E.M	% Deviation	N	Mean	S.E.M	% Deviation	N
**S**	96	1	25.9	5	157	1	17.4	5	1585	11	11.9	5
**H**	104	5	19.9	5	169	2	11.1	5	1616	11	10.2	5
**X**	131	1	0.5	5	171	1	9.8	5	1659	3	7.8	5

Table S7. Mean contact angles and standard error.

PDMS type	130 nm			190 nm			1800 nm		
	Mean	S.E.M	N	Mean	S.E.M	N	Mean	S.E.M	N
**S**	114.3	0.2	10	115.3	0.4	10	138.5	2.9	10
**H**	109.5	0.4	10	109	1.1	10	135.5	1.4	10
**X**	108.9	0.7	10	109	0.6	10	137.5	0.3	10

Table S8. Mean cell number and standard error.

PDMS type	130 nm			190 nm			1800 nm		
	Mean	S.E.M	N	Mean	S.E.M	N	Mean	S.E.M	N
**S**	54	9	10	57	4	10	38	3	10
**H**	37	3	12	45	2	12	61	7	10
**X**	77	13	11	64	2	10	61	4	10

Table S9. Mean cell area and standard error.

PDMS type	130 nm			190 nm			1800 nm		
	Mean	S.E.M	N	Mean	S.E.M	N	Mean	S.E.M	N
**S**	473	18	394	374	12	576	335	17	388
**H**	339	10	445	290	7	551	316	9	614
**X**	543	19	847	467	14	648	429	13	614

Table S10. Mean cellular elongation factor and standard error.

PDMS type	130 nm			190 nm			1800 nm		
	Mean	S.E.M	N	Mean	S.E.M	N	Mean	S.E.M	N
**S**	3.1	0.01	394	2.8	0.1	576	2.3	0.1	388
**H**	3.2	0.1	445	3.3	0.1	551	3.3	0.1	614
**X**	3.4	0.1	847	3.4	0.1	648	3.3	0.1	614
